# Perceived knowledge infrastructure capability and physicians’ job satisfaction in Chinese maternal and child health hospitals: evidence from Shenzhen

**DOI:** 10.3389/fpubh.2026.1964250

**Published:** 2026-10-23

**Authors:** Shuyan Jin, Alexandra Fernandes, Dong Wang

**Affiliations:** 1 Shenzhen Maternity and Child Healthcare Hospital, Women and Children’s Medical Center, Southern Medical University, Shenzhen, Guangdong Province, China; 2 Business Research Unit (BRU-IUL), Instituto Universitário de Lisboa (ISCTE-IUL), Lisboa, Portugal; 3 School of Health Management, Southern Medical University, Guangzhou, Guangdong Province, China

**Keywords:** China, job satisfaction, knowledge infrastructure capability, maternal and child health institutions, physician

## Abstract

**Background:**

In the knowledge economy era, effective knowledge management is crucial for healthcare institutions, especially for maternal and child health institutions (MCHIs) in China—and, in particular, in Shenzhen—that face declining fertility and a rising number of high-risk pregnancies. Improving physicians’ job satisfaction is considered important for care quality and institutional sustainability. This study aimed to examine the association of perceived knowledge infrastructure capability (KIC) with physician job satisfaction in Shenzhen MCHIs.

**Methods:**

This study comprised two components. First, a survey of 1,037 participants from 49 institutions across eastern, central, and western China was conducted to assess the psychometric properties of perceived KIC in the Chinese hospital context, using confirmatory factor analysis (CFA), Cronbach’s *α*, composite reliability, average variance extracted, and the heterotrait-monotrait ratio. A subsequent cross-sectional survey of physicians from MCHIs in Shenzhen, used structural equation modeling (SEM) to examine associations between constructs of perceived KIC and physicians’ intrinsic satisfaction (IS), extrinsic satisfaction (ES), and general satisfaction (GS).

**Results:**

The perceived KIC, comprising two constructs (i.e., knowledge governance mechanisms [KGMs] and Technology), demonstrated acceptable reliability and validity. The SEMs examining the associations between KGMs and IS, ES, and GS all showed acceptable model fit. The KGMs showed significant positive associations with IS (standardized *β* = 0.727 [95% CI: 0.553–0.901]), ES (standardized *β* = 0.679 [95% CI: 0.492–0.866]), and GS (standardized *β* = 0.724 [95% CI: 0.549–0.899]), whereas the Technology did not show statistically significant associations with these three outcomes.

**Conclusion:**

This study examined the association between perceived KIC and physicians’ job satisfaction in Shenzhen MCHIs. KGMs was significantly associated with all three job satisfaction outcomes, whereas Technology was not significantly associated with these outcomes. These findings suggest that strengthening KGMs may offer a practical approach to supporting physician job satisfaction in Shenzhen MCHIs.

## 1 Introduction

The contemporary economy has gradually shifted from industrial manufacturing to a knowledge- and service-oriented knowledge economy, with organizational operating models transitioning from resource-driven to knowledge-driven approaches. As a result, knowledge management has been widely adopted across organizations (1, 2). Knowledge infrastructure has been identified as a key component of knowledge management (3). Knowledge infrastructure capability (KIC) refers to the foundational organizational capacity that enables effective knowledge management by providing the necessary technological, structural, and cultural conditions (3–5). The healthcare sector is a typical knowledge-intensive field that relies heavily on intellectual capital and the continuous updating of medical evidence and technologies; therefore, a well-developed knowledge infrastructure is essential for enhancing institutional competitiveness and supporting the professional work of staff (6).

In recent years, declining fertility rates, together with the rising proportion of advanced-age mothers and high-risk pregnancies, have significantly altered the structure of service demand, technical complexity, and risk levels of maternal and child health institutions (MCHIs). In China, MCHIs are government-established, non-profit specialized providers with a public-health mandate that deliver preventive/public health services and basic medical care for women and children. The reduction in births has decreased service volumes, while growing demand for high-risk services has raised requirements for professional capacity and service quality, placing MCHIs under increasing survival and competitive pressure (7, 8). Shenzhen is a major megacity in southern Guangdong Province, China, with a highly developed economy and a large, mobile population. It has more than 17 million permanent residents, including about 7.89 million women, while migrants account for more than 65% of the population (9). In line with these national trends, MCHIs in Shenzhen face changing service needs and growing challenges to their organizational sustainability and competitiveness.

Job satisfaction among healthcare employees represents a critical issue in healthcare management, as it directly relates to patient safety and healthcare service quality, with potential consequences for the competitiveness of healthcare institutions (10). Job satisfaction is defined as employees’ psychological perception of the work itself and related aspects during their work process within a specific organization (11). For a long time, China’s healthcare system has placed greater emphasis on improving patient satisfaction while giving relatively insufficient attention to healthcare workers’ satisfaction (12–14). Some studies have shown that the overall level of job satisfaction among Chinese healthcare workers is relatively low and has become an important factor influencing turnover intention (15, 16). In China, nationwide surveys also indicate that clinical physicians’ satisfaction remains at a low level (17, 18).

Currently, several studies have explored the relationship between KIC and employee satisfaction across different organizational contexts. For instance, Masa’deh et al. examined the relationship between KIC and job satisfaction among university academic staff in Jordan (19). Tabejamaat et al. explored the association between KIC and job satisfaction among construction industry employees in Iran (20). In the healthcare sector, prior research has demonstrated that knowledge management practices more broadly—such as knowledge acquisition, sharing, and utilization—are positively associated with staff satisfaction (4, 5).

However, existing studies have predominantly focused on non-healthcare sectors, and limited evidence is available regarding the relationship between KIC and physician job satisfaction in Chinese hospitals. In addition, although KIC is an organizational-level concept, existing studies have commonly assessed it through employees’ individual perceptions (19, 20). Accordingly, the present study uses the term perceived KIC to explicitly reflect the individual-level nature of the measurement. Therefore, this study aims to examine the association between perceived KIC and physicians’ job satisfaction in MCHIs in Shenzhen, China.

## 2 Phase 1: measurement of perceived KIC in Chinese hospitals

### 2.1 Materials and methods

#### 2.1.1 Formation of items

Based on existing literature (3, 21, 22), this study preliminarily considered the perceived KIC dimensions (i.e., Technology, Structure, and Culture) and corresponding measurement items. These dimensions and items were initially reviewed with consideration of the Chinese hospital context and the conceptual meaning of each dimension. Subsequently, two rounds of modified Delphi consultation, incorporating semi-structured interviews and expert ratings, were conducted to obtain expert feedback and further refine the scale (23).

#### 2.1.2 Modified Delphi consultation

In selecting experts for the Delphi consultation, three criteria were considered: (1) geographical distribution (requiring experts to represent more than three different regions in China), (2) professional diversity (covering various positions related to hospitals and knowledge management), and (3) professional familiarity (experts must have sufficient knowledge of the research field) (24). A total of 18 experts were invited from Beijing, Shanghai, Shenzhen, and other regions, including hospital directors, management department heads, and researchers with expertise in hospital operations, clinical practice, health policy, and medical research. To ensure credibility, each expert completed a self-assessment form, after which expert authority coefficients were calculated, with a threshold ≥ 0.70 considered acceptable (Supplementary Method 1; Supplementary Table 1).

Two rounds of expert consultation were conducted to refine the dimensions and items. In each round, experts used a five-point Likert scale to assess the importance and appropriateness of each dimension and item, and provided open-ended feedback. Based on expert feedback from each round, expert positivity coefficient (i.e., expert responses) and degree of harmonization of expert opinions were calculated. Additionally, the mean, standard deviation (SD), and coefficient of variation (CV) of importance scores, appropriateness scores, and total scores were used to characterize experts’ evaluation of dimensions and items. The total score was calculated as the average of the importance and appropriateness scores. Based on Lian et al. (25), dimensions and items were screened as follows: items with a mean total score<4.00 and CV < 20% were considered for deletion; items with a mean total score<4.00 and CV ≥ 20% or a mean total score≥4.00 and CV ≥ 20% were retained or removed based on expert suggestions; items with a mean total score≥4.00 and CV < 20% were retained. Expert responses were deemed invalid if all item ratings were≤2.00.

#### 2.1.3 Pre-survey and focus group discussion

The Delphi-refined measurement items were used in a small-scale pre-survey to collect respondent feedback. Furthermore, expert focus group discussions were conducted to further review and refine the measurement items, ensuring their suitability for the Chinese healthcare context.

#### 2.1.4 Formal survey and data collection

A survey was specifically designed to evaluate the factor structure and psychometric properties of the scale. To determine the target sample size, an *a priori* power analysis based on the RMSEA was conducted for the CFA model (26). The analysis indicated that a minimum of 273 participants was required to achieve a statistical power of 0.80 at an alpha level of 0.05, with 41 degrees of freedom derived from the theoretical model. Allowing for 10% potentially invalid or incomplete questionnaires, the final minimum required sample size was 304 participants.

A multi-stage stratified sampling design combining typical and convenience sampling was employed to recruit participants from healthcare institutions in China. In the first stage, sampling was stratified according to geographic regions and economic development levels, covering three major regions of China: eastern, central, and western regions. In the second stage, healthcare institutions were selected through typical sampling, including both general and specialized hospitals at provincial, municipal, and county levels. Within each selected institution, healthcare professionals were categorized into four main professional groups: doctors, nurses, medical technicians, and administrative staff. Finally, convenience sampling was applied within each stratum to recruit participants who voluntarily agreed to participate.

Data were collected electronically via Wenjuanxing^1^, a widely used online survey platform in China. The questionnaire was distributed using a quick response (QR) code that participants could scan via mobile devices. To facilitate survey administration across different healthcare institutions, a designated coordinator at each participating hospital served as the contact person. The coordinators received standardized training on the research objectives and procedures and were responsible for distributing the survey QR code to eligible respondents. Participants accessed the questionnaire by scanning the QR code and completed the survey anonymously.

To assess potential common method bias (CMB) in the self-reported questionnaire data, a single-factor CFA comparison approach was adopted (27). Specifically, a single-factor model, in which all measurement items were forced to load onto a single common factor, was specified and compared with the final perceived KIC measurement model. A poor overall fit of the single-factor model, particularly when compared with the substantially better fit of the final measurement model, was interpreted as indicating that CMB was unlikely to pose a significant threat to the validity of the study.

#### 2.1.5 Reliability and construct validity assessment of the perceived KIC scale

The internal consistency of each construct was assessed using Cronbach’s alpha coefficients, with values above 0.70 considered acceptable. Construct validity was evaluated using confirmatory factor analysis (CFA), with Maximum Likelihood estimation with Robust standard errors (MLR) as the estimator. Model fit was assessed using multiple indices, including the chi-square statistic divided by degrees of freedom (χ^2^/*df*), comparative fit index (CFI), Tucker–Lewis index (TLI), root mean square error of approximation (RMSEA), and standardized root mean square residual (SRMR). Convergent validity was assessed using composite reliability (CR) and average variance extracted (AVE), with CR values above 0.70 and AVE values above 0.50 considered satisfactory. Discriminant validity was evaluated using the heterotrait–monotrait ratio of correlations (HTMT), with values below 0.85 indicating adequate discriminant validity (28).

### 2.2 Results

#### 2.2.1 Preliminary perceived KIC measurement framework

The preliminary perceived KIC scale contained three dimensions, and a total of 24 items: Technology (seven items), Structure (nine items), and Culture (eight items).

#### 2.2.2 Expert basic characteristics

A total of 18 experts were consulted. Among them, six experts were aged 50 or above, accounting for 33.33%; eight experts were aged 40–49 (44.44%); and four experts were aged 30–39 (22.22%). In terms of work experience, nine experts had more than 20 years of experience (50%), six had 15–20 years of experience (33.33%), two had 11–15 years (11.11%), and one had 6–10 years (5.56%) (Supplementary Table 2).

The expert authority coefficients ranged from 0.70 to 1.00, with a mean of 0.86, as shown in Supplementary Table 2. All 18 experts returned their assessments.

#### 2.2.3 Modified Delphi expert consultation

Based on the first round of Delphi consultation, the importance scores, appropriateness scores, overall scores, and their means, SD, and CV for each dimension and item were calculated, as detailed in Supplementary Table 3. Expert ratings of dimensions and items, as well as the resulting adjustments, are also presented in Supplementary Table 3.

Following the established screening criteria, among the 24 preliminary items, eight were deleted, two were merged, and four were optimized for clarity and wording. A second round of Delphi consultation was conducted using the refined measurement framework, and the results of this round are presented in Supplementary Table 4.

In the first round, the Kendall’s coefficient of concordance for expert ratings of the 24 items’ importance was 0.084 (*p* = 0.054), and the concordance coefficient for appropriateness ratings was 0.102 (*p* = 0.008). In the second round, Kendall’s coefficient of concordance for importance ratings of the 15 items increased to 0.278 (*p* < 0.001), and the coefficient for appropriateness ratings was 0.287 (*p* < 0.001). Detailed concordance coefficients for both rounds were provided in Supplementary Table 5.

After two rounds of Delphi expert consultation, the perceived KIC scale was refined, consisting of three dimensions and 15 items (Supplementary Table 6).

#### 2.2.4 Pre-survey and focus group discussion

Through convenience sampling, 20 hospital staff members were selected to participate in a small-scale pre-survey. During the pre-survey, respondents were individually asked whether they understood each measurement item and whether their interpretations were consistent with the intended meaning. Subsequently, focus group discussions were conducted with five experts, including two hospital administrators, one health policy researcher, and two health management researchers.

The dimensions of the perceived KIC scale were refined through a comprehensive consideration of various forms of evidence to ensure its applicability and validity within the specific context of Chinese hospitals. First, the semi-structured interviews conducted as part of the modified Delphi consultation indicated that experts generally viewed organizational structure and culture as closely interconnected rather than independent elements, jointly influencing knowledge processes in Chinese organizational contexts. Second, feedback from the pre-survey supported this perspective, as the majority of respondents reported conceptual overlap and difficulties in clearly distinguishing between items related to organizational structure and culture. Third, focus group discussions with experts further reinforced this understanding. Experts highlighted that, in Chinese management practices, cultural factors deeply influence the implementation and adaptation of formal structures and institutional arrangements, while formal structures simultaneously shape and reinforce organizational culture. Therefore, integrating these two dimensions was considered more appropriate for reflecting the realities of Chinese management contexts. Furthermore, this integration was supported by existing theoretical perspectives. Organizational theories suggest that structure and culture are interrelated and jointly shape organizational behaviors (29, 30). Particularly in the Chinese management context, formal institutions and informal relationships and cultural norms are often highly embedded, collectively shaping organizational governance mechanisms (31). In this regard, the concept of knowledge governance mechanisms (KGMs) (29, 32), provides a suitable conceptual foundation. KGMs are understood as the various formal and informal arrangements and mechanisms that organizations deploy to influence knowledge processes such as creation, sharing, and retention. Based on these multiple sources of evidence, the Structure and Culture dimensions were consolidated into a single integrated dimension termed KGMs, with the item refined accordingly.

In addition, four items were removed following further refinement based on pre-survey feedback and expert discussions, primarily due to conceptual overlap or limited relevance to knowledge-oriented characteristics. The final perceived KIC scale comprised two dimensions — Technology and KGMs — with a total of 11 items (Table 1).

**Table 1:** Dimensions and items of the final perceived KIC scale.

Dimension	No.	Item
Technology	T1	My hospital has established an effective knowledge management system to collect, categorize, store and retrieve knowledge.
	T2	My hospital has the hardware, software, networks and databases to support collaboration among staff.
	T3	My hospital has the hardware, software, networks and databases in place to support collaboration between staff and external parties.
KGMs	K1	My hospital has a system for training and continuing education.
	K2	My hospital has systems in place for academic exchanges and collaborations.
	K3	My hospital has established incentives for innovation (e.g., invention incentives, research incentives, incentives to develop new technologies and projects).
	K4	My colleagues understand the importance of knowledge in the development of the hospital.
	K5	My colleagues expect a high level of involvement in the acquisition, translation and application of knowledge.
	K6	My hospital places a strong emphasis on staff training and learning.
	K7	My colleagues are rewarded, recognized or offered better career prospects for their individual expertise.
	K8	In my hospital, senior management explicitly emphasizes the role of knowledge in the hospital’s development.

#### 2.2.5 Reliability and validity of the perceived KIC scale

In the formal survey, a total of 1,037 valid questionnaires were included, with participants recruited from 49 healthcare institutions across 10 provinces/autonomous regions and 15 cities in eastern, central, and western China.

A CFA was conducted to evaluate the psychometric properties and factor structure of the perceived KIC scale in the formal survey. The results indicated an acceptable model fit: χ^2^/*df* = 3.752, *p* < 0.001, CFI = 0.980, TLI = 0.973, RMSEA = 0.052, and SRMR = 0.050. All standardized factor loadings exceeded 0.6 (Supplementary Table 7). Cronbach’s α coefficients were 0.849 for Technology and 0.975 for KGMs. CR values for both dimensions exceeded the recommended threshold of 0.70. The AVE values were 0.665 for Technology and 0.824 for KGMs, both exceeding the recommended threshold of 0.50. The HTMT value between Technology and KGMs was 0.706, which was below the recommended threshold of 0.85. These results indicate that the perceived KIC scale demonstrated acceptable reliability and validity.

The single-factor model showed a markedly poorer fit (Supplementary Table 8), suggesting that a single factor was unlikely to adequately account for the observed covariation among the items.

## 3 Phase 2: survey of perceived KIC and physicians’ job satisfaction in Shenzhen MCHIs, China

### 3.1 Materials and methods

#### 3.1.1 Measures

##### 3.1.1.1 Demographic and occupational characteristics

The demographic and occupational characteristics assessed in this study included gender, age, education level, years of service, professional title, administrative position, monthly income, night shifts per month, and weekly working hours.

##### 3.1.1.2 Measurement of perceived KIC and job satisfaction

Perceived KIC was measured using the scale constructed in phase 1.

The Minnesota Satisfaction Questionnaire (MSQ) is among the most widely used instruments for measuring job satisfaction. Its 20-item Short Form assesses three aspects of job satisfaction: intrinsic satisfaction (IS), extrinsic satisfaction (ES), and general satisfaction (GS) (33). IS reflects employees’ satisfaction with the content and nature of the work itself and the rewards inherent in performing the job, including opportunities to use one’s abilities, achieve a sense of accomplishment, exercise creativity and responsibility, and experience variety. ES reflects satisfaction with aspects of the work environment that are external to the job content, including compensation, company policies, opportunities for advancement, supervision, and working conditions. GS provides an overall assessment of job satisfaction. The IS score is calculated as the sum of the 12 intrinsic items, the ES score as the sum of the six items, and the GS score as the sum of all 20 items. The specific items used to calculate the three job satisfaction scores are presented in Supplementary Table 9. The Cronbach’s α coefficients for the IS and ES subscales were 0.949 and 0.937, respectively, and that for the GS scale was 0.971, indicating high internal consistency reliability.

Each item of perceived KIC and job satisfaction was rated on a five-point Likert scale, ranging from 1 = very dissatisfied to 5 = very satisfied.

#### 3.1.2 Study design, sample size estimation and sampling

A cross-sectional survey was conducted at MCHIs in Shenzhen, China. For the SEM analysis, an *a priori* power analysis based on the RMSEA was conducted. With an alpha level of 0.05, a statistical power of 0.80, and 50 degrees of freedom derived from the theoretical model, the minimum required sample size was 243 participants. Allowing for 10% potentially invalid or incomplete questionnaires, the final minimum required sample size was 270 participants.

Shenzhen has a total of 11 MCHIs, among which three institutions were selected as study sites based on differences in institutional size, stage of development, and volume of diagnostic and treatment services. The selected institutions were designated as Institutions 1, 2, and 3, respectively (Supplementary Table 10). Convenience sampling was employed within the selected institutions to recruit physicians to participate in the survey. The data collection procedure was the same as that described in Section 2.1.4: participants completed the questionnaires online via the Wenjuanxing platform using QR codes distributed by institutional coordinators.

#### 3.1.3 Theoretical framework and hypothesized model

*H1a*: Technology is positively associated with IS among physicians in MCHIs in Shenzhen, China.

*H1b*: KGMs is positively associated with IS among physicians in MCHIs in Shenzhen, China.

*H2a*: Technology is positively associated with ES among physicians in MCHIs in Shenzhen, China.

*H2b*: KGMs is positively associated with ES among physicians in MCHIs in Shenzhen, China.

*H3a*: Technology is positively associated with GS among physicians in MCHIs in Shenzhen, China.

*H3b*: KGMs is positively associated with GS among physicians in MCHIs in Shenzhen, China.

#### 3.1.4 Statistical analysis

Descriptive statistics were reported as frequencies and percentages for categorical variables and means ± standard deviations (SD) for continuous variables. Group differences in IS, ES, and GS were assessed using independent-samples t-tests, Welch’s t-tests, or Mann–Whitney U tests for two-group comparisons, and one-way ANOVA, Welch’s ANOVA, or Kruskal–Wallis tests for comparisons involving three or more groups, with appropriate post-hoc tests when the overall test was significant.

To assess potential CMB, we compared a single-factor model with the final two-factor model, as described in Section 2.1.4. Reliability and validity were evaluated following the procedures described in Section 2.1.5. CFA was conducted, and Cronbach’s *α*, CR, AVE, and HTMT values were calculated and reported.

Structural equation modeling (SEM) was performed using MLR estimator to separately examine the associations between the two constructs of perceived KIC (i.e., KGMs and Technology) and GS, IS, and ES. Demographic and occupational characteristics associated with statistically significant differences in the corresponding job satisfaction outcomes in the group comparisons were included as covariates in the SEM analyses.

Model fit for both the CFA and SEM was evaluated using (χ^2^/*df*, CFI, TLI, RMSEA, and SRMR. The significance level was set at *p* < 0.05. All analyses were completed using R 4.5.0.

### 3.2 Results

#### 3.2.1 Participant characteristics and job satisfaction scores

A total of 366 valid questionnaires were included in the analysis, with 231, 101, and 34 from the three institutions, respectively. Of these participants, 277 (75.7%) were female. The largest proportion of participants were aged 35–44 years (45.6%), had 10–19 years of service (44.5%), held intermediate professional titles (41.3%), and reported a monthly income of >20,000 CNY (42.1%). The majority held no administrative position (88.5%) and worked more than 40 h per week (79.0%). More than half of the participants performed ≥4 night shifts per month (51.9%), and 58.5% held a postgraduate degree.

With respect to job satisfaction scores, statistically significant differences in IS, ES and GS scores were observed across age, professional title, years of service, monthly income, and weekly working hours (Table 2). Furthermore, post-hoc pairwise comparisons for variables with three or more groups revealed statistically significant differences between specific groups (Supplementary Table 11). Specifically, statistically significant differences were found between the <35 and 35–44 age groups, the junior or below and associate senior title groups, the <10 and 10–19 years of service groups, and the ≤15,000 CNY and >20,000 CNY monthly income groups (all adjusted *p* < 0.05).

**Table 2:** Demographic and occupational characteristics of participants and comparisons of IS, ES, and GS scores.

Factors	N (%)	IS		ES		GS	
		$\bar{X}$ ± SD	*p*	$\bar{X}$ ± SD	*p*	$\bar{X}$ ± SD	*p*
Gender			0.136		0.213		0.190
Female	277 (75.7)	45.44 ± 7.73		21.79 ± 4.72		74.97 ± 13.38	
Male	89 (24.3)	46.82 ± 8.45		22.45 ± 5.34		77.11 ± 14.99	
Age (years)			0.005		0.002		0.004
<35	108 (29.5)	47.28 ± 7.51		23.15 ± 4.61		78.61 ± 13.11	
35–44	167 (45.6)	44.35 ± 7.66		21.07 ± 4.68		72.94 ± 13.22	
≥45	91 (24.9)	46.60 ± 8.50		22.15 ± 5.26		76.47 ± 14.85	
Education level			0.929		0.446		0.656
Bachelor’s degree or below	152 (41.5)	45.89 ± 8.70		22.13 ± 5.22		75.88 ± 15.03	
Postgraduate (master’s or doctoral)	214 (58.5)	45.69 ± 7.34		21.82 ± 4.62		75.21 ± 12.88	
Professional title			0.014		0.002		0.006
Junior or below	58 (15.8)	47.74 ± 7.55		23.60 ± 4.27		79.60 ± 12.75	
Intermediate	151 (41.3)	45.99 ± 8.14		22.07 ± 5.09		75.93 ± 14.21	
Associate senior	114 (31.1)	43.88 ± 7.21		20.79 ± 4.53		72.04 ± 12.64	
Senior	43 (11.7)	47.42 ± 8.58		22.40 ± 5.15		77.58 ± 14.87	
Administrative position			0.167		0.290		0.269
No administrative role	324 (88.5)	45.61 ± 8.00		21.87 ± 4.90		75.25 ± 13.90	
With administrative role	42 (11.5)	47.07 ± 7.27		22.55 ± 4.71		77.36 ± 12.99	
Years of service			0.002		<0.001		0.001
<10	112 (30.6)	47.55 ± 7.44		23.32 ± 4.45		79.05 ± 12.86	
10–19	163 (44.5)	44.12 ± 7.84		20.98 ± 4.83		72.60 ± 13.60	
≥20	91 (24.9)	46.56 ± 8.13		22.00 ± 5.10		76.29 ± 14.27	
Monthly income (CNY 10,000)			0.026		0.012		0.014
≤1.5	107 (29.2)	47.56 ± 8.91		23.15 ± 5.15		78.84 ± 15.33	
1.5–2.0	105 (28.7)	45.33 ± 7.97		21.54 ± 5.05		74.59 ± 13.92	
>2.0	154 (42.1)	44.84 ± 6.95		21.40 ± 4.43		73.78 ± 12.17	
Night shifts per month			0.146		0.426		0.253
<4	176 (48.1)	46.35 ± 8.18		22.09 ± 5.16		76.22 ± 14.35	
≥4	190 (51.9)	45.24 ± 7.66		21.83 ± 4.61		74.82 ± 13.26	
Weekly working hours			0.024		0.042		0.030
≤40 h	77 (21.0)	47.60 ± 8.21		22.95 ± 4.59		78.58 ± 13.60	
>40 h	289 (79.0)	45.29 ± 7.79		21.69 ± 4.92		74.67 ± 13.75	

IS, intrinsic satisfaction; ES, extrinsic satisfaction; GS, general satisfaction.

#### 3.2.2 Reliability and validity of the perceived KIC scale

To confirm the psychometric properties of the perceived KIC scale in the survey sample, a CFA was conducted. Model fit indices indicated acceptable fit: χ^2^/*df* = 1.261, *p* = 0.122, CFI = 0.993, TLI = 0.990, RMSEA = 0.027, and SRMR = 0.029. All standardized factor loadings exceeded 0.6 (Supplementary Table 12). Cronbach’s α coefficients were 0.861 for Technology and 0.923 for KGMs. CR values for both dimensions exceeded the recommended threshold of 0.70. The AVE values were 0.688 for Technology and 0.594 for KGMs, both exceeding the recommended threshold of 0.50. The HTMT value between Technology and KGMs was 0.841, which was below the recommended threshold of 0.85. These results indicate that the perceived KIC scale demonstrated acceptable reliability and validity.

In addition, the single-factor model showed significantly worse fit (Supplementary Table 8), suggesting that a single factor could not adequately explain the item covariation.

#### 3.2.3 Structural equation model of perceived KIC and physicians’ job satisfaction in MCHIs

Based on the group comparisons, age, professional title, years of service, monthly income, and weekly working hours, which showed statistically significant differences in job satisfaction outcomes, were included as covariates in all three SEM models for GS, IS, and ES.

Three separate SEM models were constructed to examine the associations between perceived KIC and each job satisfaction scores. For the model examining the association between perceived KIC and IS, fit indices suggested acceptable model fit (χ^2^/*df* = 1.440, *p* < 0.001, CFI = 0.973, TLI = 0.968, RMSEA = 0.035, SRMR = 0.049). The association between Technology and IS was not statistically significant, whereas KGMs was positively associated with IS (standardized *β* = 0.727 [95% CI: 0.553–0.901], *p* < 0.001, Figure 1; Supplementary Table 13).

**Figure 1:**
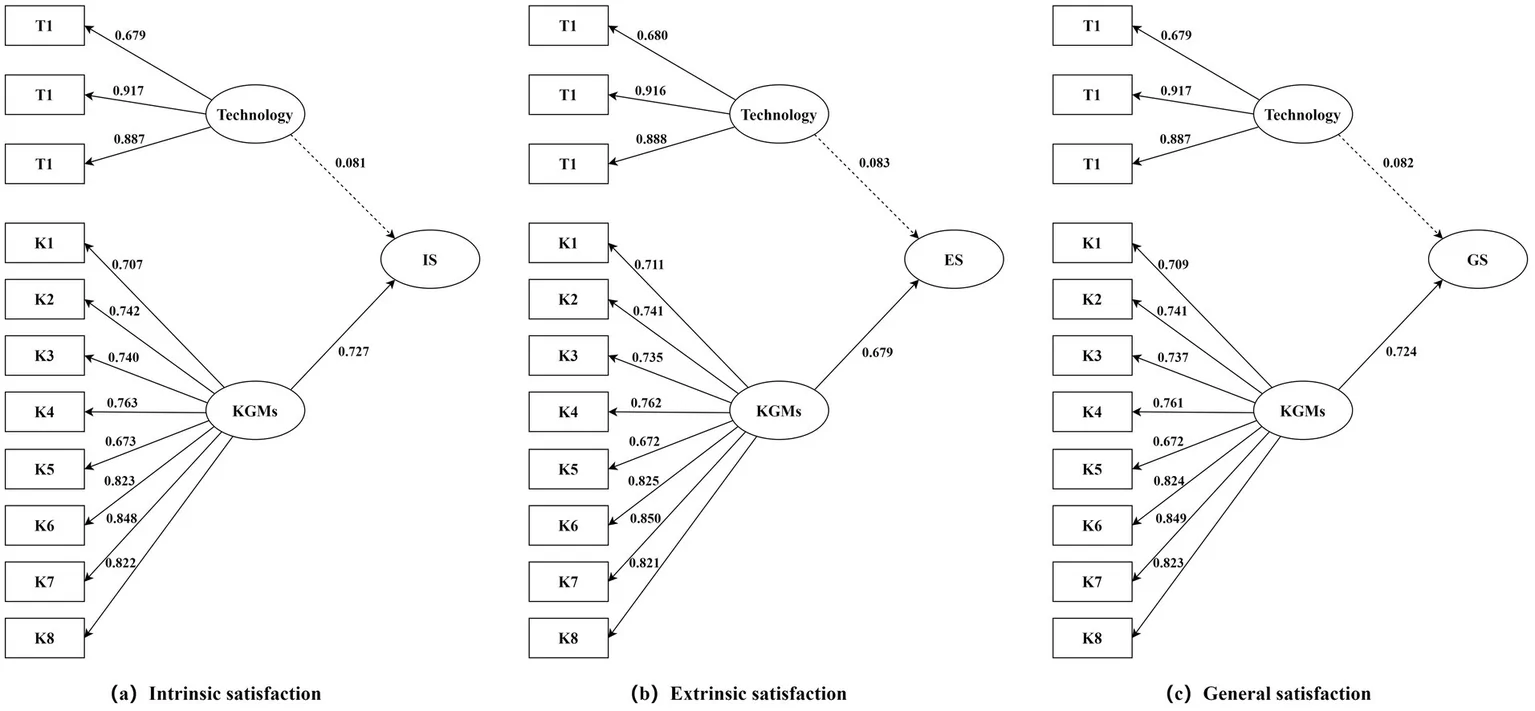
Structural equation models depicting the associations between perceived KIC and physicians’ job satisfaction. **(a)** IS model; **(b)** ES model; **(c)** GS model. Solid arrows indicate statistically significant standardized path coefficients, whereas dashed arrows indicate statistically non-significant standardized path coefficients. IS, intrinsic satisfaction; ES, extrinsic satisfaction; GS, general satisfaction; KIC, knowledge infrastructure capability; KGMs, knowledge governance mechanisms; T1–T3, items of the Technology dimension; K1–K8, items of the KGMs dimension.

For the model examining the association between perceived KIC and ES, fit indices also indicated acceptable model fit (χ^2^/*df* = 1.525, *p* < 0.001, CFI = 0.967, TLI = 0.961, RMSEA = 0.038, SRMR = 0.049). Similarly, while Technology showed no statistically significant association with ES, KGMs was positively associated with ES (standardized *β* = 0.679 [95% CI: 0.492–0.866], *p* < 0.001, Figure 1; Supplementary Table 13).

For the model examining the association between perceived KIC and GS, acceptable model fit was similarly observed (χ^2^/*df* = 1.477, *p* < 0.001, CFI = 0.970, TLI = 0.966, RMSEA = 0.036, SRMR = 0.049). The association between Technology and GS was not statistically significant, whereas KGMs was positively associated with GS (standardized *β* = 0.724 [95% CI: 0.549–0.899], *p* < 0.001, Figure 1; Supplementary Table 13).

Altogether, the findings provided empirical support for hypotheses H1b, H2b, and H3b, whereas hypotheses H1a, H2a, and H3a were not supported.

#### 3.2.4 Exploratory mediation analysis

Considering that technology may influence communication patterns, information access, and collaboration needs, it may also be associated with changes in formal structures, incentive systems, and informal cultural norms that constitute KGMs (29, 34). Therefore, an exploratory mediation analysis was further conducted to examine whether KGMs could serve as a potential pathway linking Technology to physicians’ job satisfaction.

The fit indices of the three mediation models (IS, ES, and GS) was acceptable (Supplementary Table 14). The results are presented in Figure 2 and Supplementary Table 15. The direct associations between Technology and IS, ES, and GS were not statistically significant. However, the indirect associations through Technology were statistically significant for IS (standardized *β* = 0.600 [95% CI: 0.445–0.754]), ES (standardized *β* = 0.561 [95% CI: 0.405–0.716]), and GS (standardized *β* = 0.598 [95% CI: 0.446–0.749]), as were the corresponding total associations (IS: standardized *β* = 0.680 [95% CI: 0.602–0.759]; ES: standardized *β* = 0.643 [95% CI: 0.558–0.729]; GS: standardized *β* = 0.679 [95% CI: 0.598–0.760]). These findings suggest that KGMs may serve as a potential pathway linking Technology and physicians’ job satisfaction in Shenzhen MCHIs.

**Figure 2:**
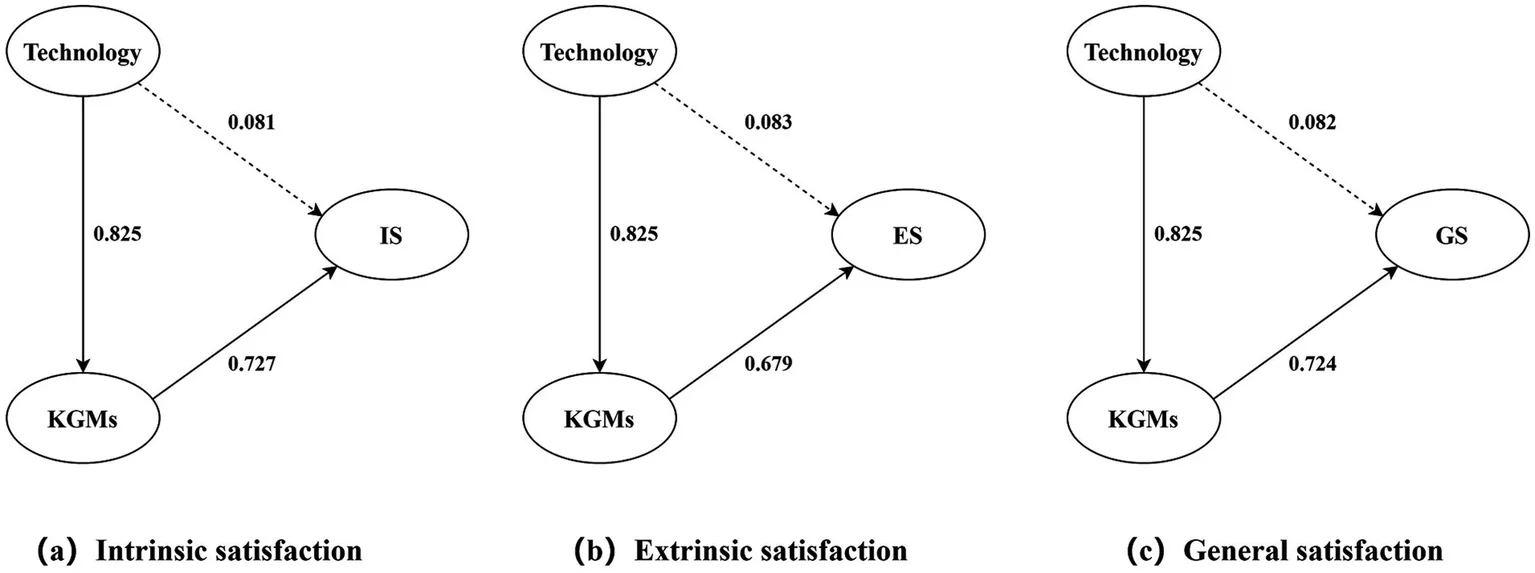
Exploratory mediation model depicting the associations between perceived KIC, KGMs, and physicians’ job satisfaction. **(a)** IS model; **(b)** ES model; **(c)** GS model. Solid arrows indicate statistically significant standardized path coefficients, whereas dashed arrows indicate statistically non-significant standardized path coefficients. KIC, knowledge infrastructure capability; KGMs, knowledge governance mechanisms; IS, intrinsic satisfaction; ES, extrinsic satisfaction; GS, general satisfaction.

## 4 Discussion

This study examined the associations between perceived KIC and physicians’ job satisfaction in Chinese MCHIs. The SEM models assessing the relationships between perceived KIC dimensions and IS, ES, and GS demonstrated acceptable overall model fit. Among the two perceived KIC dimensions, KGMs were positively associated with all three job satisfaction outcomes, whereas Technology did not show statistically significant direct associations with IS, ES, or GS. Furthermore, the exploratory mediation analysis suggested that KGMs may represent a potential pathway linking Technology and physicians’ job satisfaction.

### 4.1 The final perceived KIC structure: integrating structure and culture into KGMs

The present study identified a two-dimensional structure of perceived KIC comprising Technology and KGMs. In existing knowledge management research, Structure and Culture are typically treated as separate dimensions, reflecting a theoretical tradition that distinguishes formal organizational arrangements from informal cultural factors (35, 36). Accordingly, many existing perceived KIC measurement models assess these dimensions separately (3, 21, 22). However, findings from the scale formation process suggested that this distinction may be less applicable to the organizational context of Chinese hospitals.

In Chinese healthcare settings, formal rules and informal practices are closely intertwined through cultural norms and relational networks (37–40). Structure and Culture may therefore interact in shaping how knowledge is created, shared, and applied, rather than operating as independent domains (41, 42). This interpretation was also supported by the preliminary expert consultations and pre-survey feedback in our study. Accordingly, Structure and Culture were integrated into KGMs in the final perceived KIC scale, following the knowledge governance perspective (29, 32).

In terms of content, the KGMs dimension of the perceived KIC scale in the present study encompasses two interwoven facets of knowledge governance. Four items lean toward formal structural mechanisms: standardized training and continuing education systems (K1), structured academic exchange and collaboration platforms (K2), explicit innovation incentive schemes (K3), and organizational-level emphasis on staff learning and development (K6). These formal arrangements establish the institutional infrastructure for knowledge processes. The remaining four items tap into informal cultural and relational norms: shared recognition of knowledge value among colleagues (K4), shared expectations for active engagement in knowledge practices (K5), expertise-based recognition and career advancement (K7), and visible senior management commitment to knowledge-driven development (K8). These informal elements shape how staff perceive, participate in, and internalize knowledge practices.

### 4.2 Associations of perceived KIC with physicians’ job satisfaction

KGMs was significantly associated with IS, ES, and GS. IS reflects satisfaction with the intrinsic aspects of physicians’ work, such as opportunities to utilize their abilities, experience a sense of achievement, exercise creativity and responsibility, and engage in varied tasks. In contrast, ES reflects satisfaction with work-context factors, including organizational policies, promotion pathways, supervision, and working conditions, while GS captures overall job satisfaction. The significant associations of KGMs with both IS and ES, as well as with GS, suggest that KGMs may be relevant to physicians’ satisfaction with both the core content of their work and the broader organizational environment. This pattern is consistent with the multifaceted nature of KGMs, which encompasses professional development, knowledge-related participation, recognition, career advancement, and organizational support.

The significant association between KGMs and IS may be relevant to the formal components of KGMs. These components encompass the hospital’s systems for training and continuing education (K1), academic exchange and collaboration (K2), and innovation incentives (K3). Together, these arrangements may provide structured opportunities for skill development, peer learning, and recognition of creative and knowledge-intensive contributions. For knowledge-intensive medical professionals, whose work requires the continuous updating and application of specialized knowledge, such opportunities may be particularly relevant to aspects of work that underpin intrinsic satisfaction. Prior research suggests that developmental support, such as continuing professional education and workplace learning opportunities, may be associated with physicians’ sense of competence, autonomy, and professional belonging (43). Peer-based collaborative learning arrangements have similarly been linked to workplace satisfaction and professional growth through mutual learning and information sharing (44). In addition, formal recognition and rewards for innovation-related work in academic medical settings have been associated with greater professional fulfillment and lower levels of burnout (45).

The significant association between KGMs and ES may be related to the broader organizational context surrounding physicians’ work. The informal components of KGMs encompass colleagues’ recognition of the value of knowledge, shared expectations for participation in knowledge-related activities, the organization’s emphasis on staff training and learning, recognition and career opportunities based on individual expertise, and senior management’s explicit emphasis on the role of knowledge (K4–K8). These elements may be relevant to physicians’ perceptions of the organizational work environment, including recognition, advancement opportunities, managerial support, and interpersonal relationships. An empirical study in healthcare organizations similarly found that employees who engaged more actively in knowledge sharing tended to report better relationships with colleagues, with such participation being positively associated with job satisfaction through a greater sense of competence at work (46). A large national survey of junior doctors in the United Kingdom found that stronger perceived organizational support was associated with more positive attitudes toward the work environment (47). Evidence from performance-based recognition and compensation schemes has also indicated positive associations with physicians’ satisfaction with compensation and perceptions of fairness (48).

The significant association between KGMs and GS may reflect the broader relevance of both formal and informal components of KGMs to physicians’ overall work experiences. As KGMs encompasses arrangements related to professional development, knowledge-related participation, recognition, career advancement, and organizational support, it may be relevant to physicians’ overall evaluation of their jobs and the organizational work environment. Prior research has similarly reported significant positive associations between knowledge management practices and job satisfaction (4, 5), consistent with our findings.

The Technology items focus on knowledge management systems (T1) and technological infrastructure supporting collaboration within and beyond the hospital (T2 and T3). The exploratory mediation analysis further indicated that the association between Technology and physicians’ job satisfaction may operate through KGMs. This finding suggests that technological capability itself may not automatically translate into higher job satisfaction; rather, its value may need to be realized through corresponding organizational governance mechanisms. Prior research has noted that technological infrastructure more often functions as an enabling condition for knowledge management activities, with its actual effectiveness frequently depending on its alignment with organizational structure, management practices, and cultural environment (49, 50). In the highly specialized and knowledge-intensive organizational context of hospitals, the information access and communication capabilities afforded by Technology may need to be further embedded within KGMs before they can be translated into perceptible work support and positive work experiences for medical staff.

### 4.3 Study contributions

This study makes the following contributions. First, this study offers a perceived KIC measurement for the Chinese hospital context by integrating the structure and culture dimensions into KGMs. This integration reflects the intertwined nature of formal governance arrangements and informal cultural norms characteristic of hospital knowledge management practices in China, and may serve as a useful reference for future measurement of perceived KIC in similar settings. Second, although prior research has examined associations between KIC and job satisfaction across various organizational settings, empirical evidence specific to physicians in Chinese MCHIs remains limited. This study addresses this gap by providing empirical evidence.

### 4.4 Practical implications

The findings of this study suggest several practical implications for Shenzhen MCHIs. The significant association between KGMs and physicians’ job satisfaction indicates that knowledge governance may be considered in relation to physicians’ broader work experiences. In practice, MCHIs may strengthen opportunities for continuing education and academic exchange, while providing appropriate support for physicians to apply and develop their professional knowledge. Establishing or improving incentives for innovation may also be relevant, particularly in settings where physicians are expected to engage in research, new technology development, or other knowledge-intensive activities. At the same time, organizational attention to knowledge should extend beyond formal arrangements. Recognition of physicians’ expertise, shared expectations for participation in knowledge-related activities, support for staff learning and development, and visible managerial emphasis on the value of knowledge may help create an organizational environment in which physicians perceive their knowledge and professional contributions as valued. Taken together, these findings suggest that MCHIs may benefit from addressing both the formal arrangements and the organizational environment surrounding knowledge-related activities when considering ways to support physician job satisfaction.

Furthermore, the exploratory mediation analysis indicated an indirect association between Technology and physician job satisfaction through KGMs. Although this finding is based on cross-sectional data, it suggests that technological development may be considered together with the organizational arrangements that support learning, knowledge exchange, and innovation. When introducing or expanding technological resources, MCHIs may also consider whether physicians have adequate opportunities for training, knowledge sharing, academic collaboration, and innovation support to support their use of these resources. This integrated perspective may provide a more useful basis for aligning technological development with physicians’ professional and organizational needs.

### 4.5 Limitations

This study has several limitations. First, the empirical data for the application study were collected from only three MCHIs in Shenzhen, which limits the generalizability of the findings to other regions or healthcare settings. Second, the study focused exclusively on physicians, and caution should be exercised when extrapolating the findings to other healthcare professionals, such as nurses and medical technicians. Third, although common method bias was assessed and no substantial concern was identified, all variables were collected from the same respondents, and the possibility of common method variance cannot be completely excluded. Fourth, the application study used a cross-sectional design; therefore, the observed associations do not establish temporal or causal relationships. Moreover, the first round of the Delphi consultation showed a relatively low level of consensus, which may have reflected the broad scope of the perceived KIC concept and the multidisciplinary backgrounds of the participating experts. Before the second round, the results and comments from the first round were summarized and fed back to all participating experts, allowing them to consider the collective views when re-evaluating the items and facilitating greater consensus in the subsequent round. Finally, the small number of participating institutions and the unbalanced institution-level sample sizes limited the feasibility of conducting robust multilevel analyses.

Future research may consider expanding the study scope by including MCHIs from multiple regions across China and extending the investigation to other types of hospitals to enhance the generalizability of the findings. Incorporating diverse healthcare professional groups would also provide a more comprehensive understanding of the association between perceived KIC and job satisfaction in healthcare settings. Future studies could further include a larger number of institutions with more balanced institution-level sample sizes and use longitudinal or other appropriate designs to examine the temporal relationships between perceived KIC and job satisfaction.

## 5 Conclusion

This study examined the association between perceived KIC and physicians’ job satisfaction in Shenzhen MCHIs. KGMs was significantly associated with all three job satisfaction outcomes, whereas Technology was not significantly associated with these outcomes. These findings suggest that strengthening KGMs may offer a practical approach to supporting physician job satisfaction in Shenzhen MCHIs.

## Data Availability

The datasets presented in this article are not readily available because the datasets used in this study are available from the authors upon reasonable request. Requests to access the datasets should be directed to SJ (13823373712@163.com).

## Footnotes

1
https://www.wjx.cn/
