# Supplementary material for: Perceived knowledge infrastructure capability and physicians’ job satisfaction in Chinese maternal and child health hospitals: evidence from Shenzhen
Source: Front Public Health. 2026 Oct 23;14:1964250. doi: 10.3389/fpubh.2026.1964250 (PMC13739786; doi:10.3389/fpubh.2026.1964250)
Supplement: Supplementary file 1 [file Data_Sheet_1.docx]

Supplementary Material

**Supplementary Method 1.** The calculation of expert authority coefficient.

The expert authority coefficient (Cr) was calculated using the formula Cr = (Ca + Cs)/2, where Ca represents the judgment coefficient and Cs denotes the familiarity coefficient. Familiarity was categorized into five levels: very familiar (1.0), familiar (0.8), average (0.5), less familiar (0.2), and very unfamiliar (0). The judgment coefficient Ca was based on expert’s assessment of the influence of theoretical analysis, theoretical analysis, practical experience, peer understanding, and intuition, each rated on a scale of large, medium and small. A quantitative table was used to assign specific values, as shown in **Supplementary** **Table 1**. The expert authority coefficient was calculated for each expert, with a value of ≥ 0.70 considered acceptable.

**Supplementary Table 1.** Quantitative table of the basis for the expert’s judgment on the indicator.

| **Basis of judgment** | **The extent to which the basis of judgment influences the expert’s judgment** | | |
| --- | --- | --- | --- |
|  | **Large** | **Medium** | **Small** |
| Theoretical analysis | 0.3 | 0.2 | 0.1 |
| Practical experience | 0.5 | 0.4 | 0.3 |
| Peer understanding | 0.1 | 0.1 | 0.1 |
| Intuition | 0.1 | 0.1 | 0.1 |

**Supplementary Table 2.** Delphi expert consultant profiles.

| **N** | **Age** | **E^*^** | **S^*^** | **Working**  **experience**  **(years)** | **Familiarity**  **factor** | **Expert judgment factor self-assessment** | | | | **Authority factor** |
| --- | --- | --- | --- | --- | --- | --- | --- | --- | --- | --- |
|  |  |  |  |  |  | **Theoretical analysis** | **Practical experience** | **Peer understanding** | **Expert intuition** |  |
| 1 | ≥40 and <50 | 2 | 1 | >20 | 0.80 | 0.20 | 0.50 | 0.10 | 0.10 | 0.85 |
| 2 | ≥50 | 3 | 1 | >20 | 0.80 | 0.30 | 0.40 | 0.10 | 0.10 | 0.85 |
| 3 | ≥50 | 2 | 2 | >20 | 0.80 | 0.30 | 0.40 | 0.10 | 0.10 | 0.85 |
| 4 | ≥40 and <50 | 3 | 3 | >20 | 0.80 | 0.20 | 0.40 | 0.10 | 0.10 | 0.80 |
| 5 | ≥50 | 2 | 3 | >20 | 0.80 | 0.20 | 0.40 | 0.10 | 0.10 | 0.80 |
| 6 | ≥40 and <50 | 2 | 4 | >20 | 0.80 | 0.20 | 0.50 | 0.10 | 0.10 | 0.85 |
| 7 | ≥30 and <40 | 2 | 4 | 11-15 | 0.80 | 0.20 | 0.40 | 0.10 | 0.10 | 0.80 |
| 8 | ≥50 | 3 | 5 | >20 | 0.80 | 0.30 | 0.40 | 0.10 | 0.10 | 0.85 |
| 9 | ≥40 and <50 | 2 | 6 | 15-20 | 1.00 | 0.30 | 0.50 | 0.10 | 0.10 | 1.00 |
| 10 | ≥30 and <40 | 2 | 7 | 6-10 | 0.50 | 0.20 | 0.50 | 0.10 | 0.10 | 0.70 |
| 11 | ≥40 and <50 | 2 | 7 | 15-20 | 0.80 | 0.20 | 0.40 | 0.10 | 0.10 | 0.80 |
| 12 | ≥40 and <50 | 2 | 7 | 15-20 | 1.00 | 0.20 | 0.50 | 0.10 | 0.10 | 0.95 |
| 13 | ≥30 and <40 | 2 | 8 | 15-20 | 0.50 | 0.30 | 0.50 | 0.10 | 0.10 | 0.75 |
| 14 | ≥50 | 1 | 8 | >20 | 0.80 | 0.30 | 0.50 | 0.10 | 0.10 | 0.90 |
| 15 | ≥40 and <50 | 3 | 9 | 15-20 | 1.00 | 0.30 | 0.50 | 0.10 | 0.10 | 1.00 |
| 16 | ≥30 and <40 | 3 | 10 | 11-15 | 0.80 | 0.30 | 0.40 | 0.10 | 0.10 | 0.85 |
| 17 | ≥40 and <50 | 3 | 10 | 15-20 | 1.00 | 0.30 | 0.50 | 0.10 | 0.10 | 1.00 |
| 18 | ≥50 | 3 | 11 | >20 | 0.80 | 0.20 | 0.50 | 0.10 | 0.10 | 0.85 |

E^*^: Education 1=undergraduate; 2=bachelor; 3=doctorate

S^*^: Specialization 1=strategic management of hospitals; 2=hospital operations management; 3=hospital informatization management; 4=hospital research and teaching management; 5=patient case statistics; 6=healthcare quality management; 7=clinical medicine; 8=nursing care; 9=health policy research; 10=health management research; 11=medical research.

**Supplementary Table 3**. Expert ratings and adjustment results in the first Delphi round for scale dimensions and items.

| **Dimension or entry** | **Importance** | | | **Appropriateness** | | | **Overall** | | | **Changes in dimensions and entries after the first round of Delphi expert consultation** |
| --- | --- | --- | --- | --- | --- | --- | --- | --- | --- | --- |
|  | **Mean** | **SD** | **CV** | **Mean** | **SD** | **CV** | **Mean** | **SD** | **CV** |  |
| Technology | 4.56 | 0.62 | 13.52% | 4.39 | 0.61 | 13.85% | 4.47 | 0.55 | 12.41% | Reservations |
| Structure | 4.56 | 0.62 | 13.52% | 4.33 | 0.59 | 13.71% | 4.44 | 0.51 | 11.50% | Reservations |
| Culture | 4.39 | 0.78 | 17.72% | 4.39 | 0.61 | 13.85% | 4.39 | 0.65 | 14.91% | Reservations |
| Q1 | 4.33 | 0.84 | 19.39% | 3.94 | 0.87 | 22.12% | 4.14 | 0.78 | 18.90% | Reservations |
| Q2 | 4.17 | 0.62 | 14.84% | 4.11 | 0.76 | 18.45% | 4.14 | 0.66 | 15.94% | Reservation, optimized expression |
| Q3 | 4.00 | 0.59 | 14.85% | 4.11 | 0.68 | 16.45% | 4.06 | 0.59 | 14.58% | Reserved, optimized expression |
| Q4 | 3.94 | 0.87 | 22.12% | 3.72 | 0.96 | 25.74% | 3.83 | 0.87 | 22.81% | Merged with entry 5 |
| Q5 | 4.00 | 0.84 | 21.00% | 3.78 | 0.94 | 24.96% | 3.89 | 0.85 | 21.85% | Merge with entry 4 |
| Q6 | 4.11 | 0.68 | 16.45% | 4.17 | 0.79 | 18.86% | 4.14 | 0.70 | 16.99% | Reserve |
| Q7 | 3.89 | 0.76 | 19.50% | 3.56 | 0.92 | 25.92% | 3.72 | 0.71 | 19.12% | Delete |
| Q8 | 3.94 | 0.94 | 23.77% | 3.28 | 0.83 | 25.21% | 3.61 | 0.72 | 19.90% | Delete |
| Q9 | 3.67 | 1.03 | 28.06% | 3.67 | 0.84 | 22.91% | 3.67 | 0.91 | 24.75% | Delete |
| Q10 | 4.39 | 0.70 | 15.90% | 4.28 | 0.67 | 15.64% | 4.33 | 0.62 | 14.27% | Retain |
| Q11 | 4.28 | 0.75 | 17.58% | 3.94 | 0.87 | 22.12% | 4.11 | 0.74 | 17.97% | Reservation |
| Q12 | 4.2 | 0.89 | 20.92% | 3.94 | 0.94 | 23.77% | 4.11 | 0.83 | 20.25% | Reserved, Optimized Expression |
| Q13 | 4.39 | 0.78 | 17.72% | 3.89 | 1.02 | 26.30% | 4.14 | 0.84 | 20.22% | Retain, Optimize Expression |
| Q14 | 3.94 | 0.94 | 23.77% | 3.67 | 1.08 | 29.58% | 3.81 | 0.96 | 25.15% | Delete |
| Q15 | 4.22 | 0.81 | 19.15% | 3.78 | 1.00 | 26.56% | 4.00 | 0.84 | 21.00% | Delete |
| Q16 | 3.78 | 0.88 | 23.25% | 3.72 | 0.89 | 24.04% | 3.75 | 0.79 | 21.08% | Delete |
| Q17 | 4.28 | 0.57 | 13.43% | 4.22 | 0.65 | 15.32% | 4.25 | 0.55 | 12.92% | Retain |
| Q18 | 4.28 | 0.75 | 17.58% | 4.11 | 0.68 | 16.45% | 4.19 | 0.67 | 15.91% | Reservation |
| Q19 | 4.56 | 0.62 | 13.52% | 4.39 | 0.61 | 13.85% | 4.47 | 0.55 | 12.41% | Delete |
| Q20 | 4.28 | 0.75 | 17.58% | 4.11 | 0.68 | 16.45% | 4.19 | 0.69 | 16.43% | Delete |
| Q21 | 3.78 | 0.73 | 19.38% | 3.50 | 0.92 | 26.39% | 3.64 | 0.72 | 19.89% | Delete |
| Q22 | 4.33 | 0.69 | 15.83% | 4.06 | 0.73 | 17.89% | 4.19 | 0.62 | 14.82% | Reservations |
| Q23 | 3.89 | 0.90 | 23.15% | 3.78 | 0.81 | 21.40% | 3.83 | 0.80 | 20.98% | Delete |
| Q24 | 4.28 | 0.67 | 15.64% | 4.22 | 0.65 | 15.32% | 4.25 | 0.60 | 14.12% | Reservations |

**Supplementary Table 4.** Expert ratings in the second Delphi round for scale dimensions and items.

| **Entry** | **Importance** | | | **Appropriateness** | | | **Overall** | | |
| --- | --- | --- | --- | --- | --- | --- | --- | --- | --- |
|  | **Mean** | **SD** | **CV** | **Mean** | **SD** | **CV** | **Mean** | **SD** | **CV** |
| Q1 | 4.89 | 0.32 | 6.61% | 4.06 | 0.73 | 17.89% | 4.47 | 0.32 | 7.15% |
| Q2 | 4.17 | 0.71 | 16.97% | 5.00 | 0.00 | 0.00% | 4.58 | 0.35 | 7.71% |
| Q3 | 4.11 | 0.76 | 18.45% | 4.94 | 0.24 | 4.77% | 4.53 | 0.36 | 8.01% |
| Q4 | 4.11 | 0.76 | 18.45% | 4.06 | 0.80 | 19.78% | 4.08 | 0.49 | 12.06% |
| Q5 | 4.11 | 0.76 | 18.45% | 4.89 | 0.32 | 6.61% | 4.50 | 0.42 | 9.34% |
| Q6 | 5.00 | 0.00 | 0.00% | 4.83 | 0.38 | 7.93% | 4.92 | 0.19 | 3.90% |
| Q7 | 4.94 | 0.24 | 4.77% | 4.06 | 0.73 | 17.89% | 4.50 | 0.38 | 8.52% |
| Q8 | 4.11 | 0.68 | 16.45% | 4.06 | 0.73 | 17.89% | 4.08 | 0.46 | 11.31% |
| Q9 | 4.11 | 0.68 | 16.45% | 4.06 | 0.73 | 17.89% | 4.08 | 0.43 | 10.50% |
| Q10 | 4.83 | 0.38 | 7.93% | 5.00 | 0.00 | 0.00% | 4.92 | 0.19 | 3.90% |
| Q11 | 4.94 | 0.24 | 4.77% | 4.83 | 0.51 | 10.64% | 4.89 | 0.27 | 5.61% |
| Q12 | 5.00 | 0.00 | 0.00% | 4.89 | 0.32 | 6.61% | 4.94 | 0.16 | 3.27% |
| Q13 | 5.00 | 0.00 | 0.00% | 4.89 | 0.32 | 6.61% | 4.94 | 0.16 | 3.27% |
| Q14 | 4.78 | 0.55 | 11.48% | 4.94 | 0.24 | 4.77% | 4.86 | 0.29 | 5.91% |
| Q15 | 4.28 | 0.75 | 17.58% | 5.00 | 0.00 | 0.00% | 4.64 | 0.38 | 8.10% |

**Supplementary Table 5.** Coordination coefficients and test results for two rounds of Delphi consultation.

| **Enquiry** | **Evaluation** | **Kendall’s Coefficient of Coordination (W)** | **χ²** | ***P*** |
| --- | --- | --- | --- | --- |
| First round | Importance rating | 0.084 | 34.832 | 0.054 |
|  | Appropriateness rating | 0.102 | 42.424 | 0.008 |
| Second round | Importance rating | 0.278 | 60.027 | <0.001 |
|  | Appropriateness rating | 0.287 | 62.031 | <0.001 |

**Supplementary Table 6.** Dimensions and items of the perceived KIC scale following two rounds of Delphi expert consultation.

| **Dimension** | **No.** | **Item** |
| --- | --- | --- |
| Technology | T1 | My hospital has established an effective knowledge management system to collect, categorize, store and retrieve knowledge. |
|  | T2 | My hospital has the hardware, software, networks and databases to support collaboration among staff. |
|  | T3 | My hospital has the hardware, software, networks and databases in place to support collaboration between staff and external parties. |
|  | T4 | My hospital has the technological infrastructure that enables employees at different locations to participate in the same learning or training activities. |
|  | T5 | My hospital has the technological infrastructure that enables employees to search for and acquire new knowledge in the healthcare sector. |
| Structure | S1 | My hospital has a system for training and continuing education. |
|  | S2 | My hospital has systems in place for academic exchanges and collaborations. |
|  | S3 | My hospital has established knowledge-sharing incentive systems (e.g., incentives for mentoring and training). |
|  | S4 | My hospital has established incentives for innovation (e.g., invention incentives, research incentives, incentives to develop new technologies and projects). |
| Culture | C1 | My colleagues understand the importance of knowledge in the development of the hospital. |
|  | C2 | My colleagues expect a high level of involvement in the acquisition, translation and application of knowledge. |
|  | C3 | My hospital places a strong emphasis on staff training and learning. |
|  | C4 | My colleagues are rewarded, recognized or offered better career prospects for their individual expertise. |
|  | C5 | The overall vision and goals of my hospital are clearly defined. |
|  | C6 | In my hospital, senior management explicitly emphasizes the role of knowledge in the hospital’s development. |

Abbreviations: KIC, knowledge infrastructure capability; T1–T3, items of the technological dimension; S1–S4, items of the structural dimension; C1-C6, tems of the cultural dimension.

**Supplementary Table 7.** Psychometric properties of the perceived KIC measure (N = 1,037).

| **Dimension** | **Item** | **Loading** | **Cronbach’s α** | | **CR** | **AVE** |
| --- | --- | --- | --- | --- | --- | --- |
| Technology | T1 | 0.717 | 0.849 | 0.856 | | 0.665 |
|  | T2 | 0.869 |  |  | |  |
|  | T3 | 0.880 |  |  | |  |
| KGMs | K1 | 0.905 | 0.975 | 0.969 | | 0.824 |
|  | K2 | 0.909 |  |  | |  |
|  | K3 | 0.902 |  |  | |  |
|  | K4 | 0.914 |  |  | |  |
|  | K5 | 0.882 |  |  | |  |
|  | K6 | 0.917 |  |  | |  |
|  | K7 | 0.910 |  |  | |  |
|  | K8 | 0.921 |  |  | |  |

Abbreviations: KIC, knowledge infrastructure capability; CR, composite reliability; AVE, average variance extracted; KGMs, knowledge governance mechanisms; T1–T3, items of the technological dimension; K1–K8, items of the KGMs.

**Supplementary Table 8.** Comparison of CFA model fit for assessing common method bias.

| **Model** | $\boldsymbol{\chi}\mathbf{^{2}/}\boldsymbol{df}$ | **CFI** | **TLI** | **RMSEA** | **SRMR** |
| --- | --- | --- | --- | --- | --- |
| Single-factor (Survey 1, N=1,037) | 12.101 | 0.917 | 0.891 | 0.103 | 0.071 |
| Two-factor (Survey 1, N=1,037) | 3.752 | 0.980 | 0.973 | 0.052 | 0.050 |
| Single-factor (Survey 2, N=366) | 3.441 | 0.932 | 0.910 | 0.082 | 0.043 |
| Two-factor (Survey 2, N=366) | 1.261 | 0.993 | 0.990 | 0.027 | 0.029 |

**Supplementary Table 9.** Minnesota satisfaction questionnaire short form used in this study.

| **No.** | **Item** |
| --- | --- |
| 1 | The extent to which my job keeps me busy. |
| 2 | The opportunity to work independently. |
| 3 | The chance to do different things. |
| 4 | The chance to be a member of a team. |
| 5 | The way my supervisor treats employees. |
| 6 | Managers’ competence in making decisions. |
| 7 | Being able to do things that do not violate my conscience. |
| 8 | The stable employment my job provides. |
| 9 | The opportunity to help others at work. |
| 10 | The opportunity to direct others' work. |
| 11 | The opportunity to use my abilities at work. |
| 12 | The way organizational policies are put into practice. |
| 13 | My pay in relation to the amount of work I do. |
| 14 | Opportunities for promotion. |
| 15 | The opportunity to use my own judgment. |
| 16 | The opportunity to work in my own way. |
| 17 | Working conditions and environment. |
| 18 | The way colleagues get along with each other. |
| 19 | Praise for doing a good job. |
| 20 | The sense of achievement I get from my work. |

Note. The intrinsic satisfaction score is computed as the sum of items 1, 2, 3, 4, 7, 8, 9, 10, 11, 15, 16, and 20 (12 items). The extrinsic satisfaction score is computed as the sum of items 5, 6, 12, 13, 14, and 19 (6 items). The general satisfaction score is computed as the sum of all 20 items.

**Supplementary Table 10.** Basic information about the three maternal and child health hospitals.

| **Hospital code** | **Year established** | **Building area (10,000 square meters)** | **Number of physicians** |
| --- | --- | --- | --- |
| 1 | 1979 | 14 | 821 |
| 2 | 1993 | 7.89 | 358 |
| 3 | 2013 | 1.40 | 123 |

**Supplementary Table 11**. Pairwise post-hoc comparisons of IS, ES, and GS across factors groups.

| **Factor** | **Group 1** | **Group 2** | **Adjusted *P*（IS）** | **Adjusted *P*（ES）** | **Adjusted *P*（GS）** |
| --- | --- | --- | --- | --- | --- |
| Age (years) | <35 | 35-44 | 0.010 | 0.002 | 0.004 |
|  | <35 | ≥45 | 1.000 | 0.602 | 1.000 |
|  | 35-44 | ≥45 | 0.056 | 0.177 | 0.113 |
| Professional title | Junior or below | Intermediate | 0.936 | 0.261 | 0.489 |
|  | Junior or below | Associate senior | 0.026 | 0.001 | 0.005 |
|  | Junior or below | Senior | 1.000 | 1.000 | 1.000 |
|  | Intermediate | Associate senior | 0.313 | 0.127 | 0.194 |
|  | Intermediate | Senior | 1.000 | 1.000 | 1.000 |
|  | Associate senior | Senior | 0.122 | 0.269 | 0.223 |
| Working years | <10 | 10-19 | 0.002 | 0.001 | 0.001 |
|  | <10 | ≥20 | 1.000 | 0.190 | 0.533 |
|  | 10-19 | ≥20 | 0.084 | 0.400 | 0.170 |
| Monthly income | ≤1.5 | 1.5-2.0 | 0.141 | 0.085 | 0.104 |
|  | ≤1.5 | >2.0 | 0.027 | 0.013 | 0.014 |
|  | 1.5-2.0 | >2.0 | 1.000 | 1.000 | 1.000 |

**Supplementary Table 12.** Confirmatory factor analysis of the perceived KIC measure in the survey sample (N = 366).

| **Construct** | **Item** | **Loading** | **Cronbach’s α** | | **CR** | **AVE** |
| --- | --- | --- | --- | --- | --- | --- |
| Technology | T1 | 0.678 | 0.861 | 0.867 | | 0.688 |
|  | T2 | 0.918 |  |  | |  |
|  | T3 | 0.887 |  |  | |  |
| KGMs | K1 | 0.708 | 0.923 | 0.902 | | 0.594 |
|  | K2 | 0.741 |  |  | |  |
|  | K3 | 0.746 |  |  | |  |
|  | K4 | 0.780 |  |  | |  |
|  | K5 | 0.685 |  |  | |  |
|  | K6 | 0.820 |  |  | |  |
|  | K7 | 0.843 |  |  | |  |
|  | K8 | 0.812 |  |  | |  |

Abbreviations: KIC, knowledge infrastructure capability; CR, composite reliability; AVE, average variance extracted; KGMs, knowledge governance mechanisms; T1–T3, items of the technological dimension; K1–K8, items of the KGMs.

**Supplementary Table 13. Standardized structural path coefficients from SEM.**

| **Hypothesis** | **From** | **To** | **Standardized *β* (95% CI)** | **SE** | **Z** | ***P*** | **Decision** |
| --- | --- | --- | --- | --- | --- | --- | --- |
| H1a | Technology | IS | 0.081 (-0.112, 0.274) | 0.098 | 0.818 | 0.413 | Not Supported |
| H1b | KGMs | IS | 0.727 (0.553, 0.901) | 0.089 | 8.179 | < 0.001 | Supported |
| H2a | Technology | ES | 0.083 (-0.119, 0.284) | 0.103 | 0.802 | 0.422 | Not Supported |
| H2b | KGMs | ES | 0.679 (0.492, 0.886) | 0.095 | 7.129 | < 0.001 | Supported |
| H3a | Technology | GS | 0.082 (-0.112, 0.275) | 0.099 | 0.828 | 0.408 | Not Supported |
| H3b | KGMs | GS | 0.724 (0.549, 0.899) | 0.089 | 8.120 | < 0.001 | Supported |

Abbreviations: IS, intrinsic satisfaction; ES, extrinsic satisfaction; GS, general satisfaction; KGMs, knowledge governance mechanisms.

**Supplementary Table 14.** Model fit indices for the structural equation model with the mediation path.

| **Outcome** | $\boldsymbol{\chi}\mathbf{^{2}/}\boldsymbol{df}$ | ***P*** | **CFI** | **TLI** | **RMSEA** | **SRMR** |
| --- | --- | --- | --- | --- | --- | --- |
| IS | 1.440 | <0.001 | 0.973 | 0.968 | 0.035 | 0.049 |
| ES | 1.525 | <0.001 | 0.967 | 0.961 | 0.038 | 0.049 |
| GS | 1.477 | <0.001 | 0.970 | 0.966 | 0.036 | 0.049 |

**Supplementary Table 15.** Results of exploratory mediation analysis

| **Outcome** | **Path** | **Standardized *β* (95% CI)** | ***P*** |
| --- | --- | --- | --- |
| IS | Technology → KGMs | 0.825 (0.770, 0.881) | <0.001 |
|  | KGMs → IS | 0.727 (0.553, 0.901) | <0.001 |
|  | Indirect effect:Technology → KGMs → IS | 0.600 (0.445, 0.754) | <0.001 |
|  | Direct effect: Technology → IS | 0.081 (-0.112, 0.274) | 0.413 |
|  | Total effect: Technology → IS | 0.680 (0.602, 0.759) | <0.001 |
| ES | Technology → KGMs | 0.825 (0.770, 0.881) | <0.001 |
|  | KGMs → ES | 0.679 (0.492, 0.866) | <0.001 |
|  | Indirect effect: Technology → KGMs → ES | 0.561 (0.405, 0.716) | <0.001 |
|  | Direct effect: Technology → ES | 0.083 (-0.119, 0.284) | 0.422 |
|  | Total effect: Technology → ES | 0.643 (0.558, 0.729) | <0.001 |
| GS | Technology → KGMs | 0.825 (0.770, 0.881) | <0.001 |
|  | KGMs → GS | 0.724 (0.549, 0.899) | <0.001 |
|  | Indirect effect: Technology → KGMs → GS | 0.598 (0.446, 0.749) | <0.001 |
|  | Direct effect: Technology → GS | 0.082 (-0.112, 0.275) | 0.408 |
|  | Total effect: Technology → GS | 0.679 (0.598, 0.760) | <0.001 |

Abbreviations: IS, intrinsic satisfaction; ES, extrinsic satisfaction; GS, general satisfaction; KGMs, knowledge governance mechanisms.
